# Synergistic immuno-modulatory activity in human macrophages of a medicinal mushroom formulation consisting of Reishi, Shiitake and Maitake

**DOI:** 10.1371/journal.pone.0224740

**Published:** 2019-11-07

**Authors:** Brody Mallard, David N. Leach, Hans Wohlmuth, Joe Tiralongo

**Affiliations:** 1 Institute for Glycomics, Griffith University, Gold Coast, Queensland, Australia; 2 Integria Healthcare, Eight Mile Plains, Queensland, Australia; 3 National Institute of Complementary Medicine, Western Sydney University, Penrith, New South Wales, Australia; 4 School of Chemistry & Molecular Biosciences, The University of Queensland, St Lucia, Queensland, Australia; City of Hope, UNITED STATES

## Abstract

A key characteristic of mushroom polysaccharides that elicit an immunomodulatory response is that they are rich in β-glucans and low in α-glucans. In this study we analysed nine commercially available preparations from three mushroom species, Reishi (*Ganoderma lucidum*), Shiitake (*Lentinula edodes*) and Maitake (*Grifola frondosa*), for β- and α-glucan content. Based on β- and α-glucan content we selected three extracts to combine into a formula and evaluated the ability of the individual extracts and formula to impact on the expression of cytokines IL-1α, IL-6, IL-10 and TNF-α in human macrophages with and without LPS stimulation. The majority of mushroom extracts and the formula were found to be highly potent immuno-stimulators possessing EC_50_ values lower than 100 μg/mL. Interestingly the mushroom formula had lower EC_50_ values in TNF-α expression from LPS stimulated macrophages compared to the individual extracts, suggesting a potential synergistic effect of the mushroom formula. A response additivity graph and curve-shift analysis illustrated that indeed the mushroom formula exhibited an immuno-stimulatory synergistic effect on the expression of the majority of cytokines evaluated in both LPS stimulated and non-stimulated human macrophages, with IL-10 having an antagonistic response. This study represents the first report of a synergistic immuno-modulatory response in human macrophages elicited from a mushroom formula rationally derived from β- and α-glucan content.

## Introduction

Medicinal mushrooms possess a variety of bioactive compounds with immunomodulatory activities, such as polysaccharides, polysaccharopeptides, phenolic compounds, proteins, lipid components and terpenoids [1]. These immunomodulatory effects and subsequent potential for therapeutic benefits are generating a renewed interest in the scientific investigation of medicinal mushrooms, represented in the market by continual steady increases in worldwide sales [2, 3]. Mushroom polysaccharides, particularly β-D-glucans, are the focus of much of this interest.

Approximately 80% of the mushroom cell wall consists of polysaccharides, with about half of these being β-glucans. Glucans are the common name given to a group of chemically heterogeneous glucose (Glc) based polysaccharides that are classified based on the nature of the glycosidic linkage as either α- or β-glucans. β-Glucans have a common structure comprising a main chain of β-(1,3)- and/or β-(1,4)-D-Glc units, along with side chain Glc of varying lengths and linkage [4]. What distinguishes fungal β-glucans is the presence of β-(1,6)-glucopyranosidic side chains. β-Glucans, specifically branched (1,3:1,6) β-glucans of fungal origin are potent immunological stimulators in humans. β-Glucans are not synthesized by humans, so these compounds are recognized by the immune system as non-self molecules, inducing both innate and adaptive immune responses [5], thus offering protection from attack by pathogenic microbes and from harmful effects of environmental toxins and carcinogens.

There is extensive literature supporting the immune modulating effect of β-glucans *in vivo*. The extent to which β-glucans are absorbed whole from the digestive tract is not fully resolved, however it is likely that the principal site of interaction with immune cells is in gut-associated lymphoid tissue. Both macrophages and dendritic cells present at gut mucosal surfaces have glucan-binding surface receptors capable of activating signal transduction pathways that result in modulation of immune responses [6–9].

A key characteristic of mushroom polysaccharides that elicit an immunomodulatory response is that they are rich in β-glucans and low in α-glucans (β- and α-glucan content typically 10–40% and <5%, respectively [10]). In this study we analysed nine commercially available preparations from three mushroom species previously reported to stimulate the immune system [5, 11], Reishi (*Ganoderma lucidum*), Shiitake (*Lentinula edodes*) and Maitake (*Grifola frondosa*), for β- and α-glucan content. Based on their β- and α-glucan content we selected three extracts to combine into a formula, with the immunostimulatory effect of the individual extracts and the formula being assessed for their ability to impact the expression of cytokines in human macrophages.

## Materials and methods

### Mushroom preparations

Mushroom preparations, including extracts of fruiting bodies and preparations containing mycelium as well as fruiting bodies (Table 1), were obtained from various standard commercial suppliers and outlets as outlined in the International PCT Application No. PCT/AU2019/050295 [12]. Briefly, these commercial mushroom extracts are prepared by the suppliers using standard methods from whole dried mushrooms grown in China. Briefly, the ground mushroom was extracted by repeated decoction (2–3hr at 80°C) and the final solution centrifuged to remove residues. The final extract was concentrated under vacuum at 55°C and the concentrate spray-dried for 0.5–1 sec at 180°C. Commercially available extracts generated in this way were obtained from Nammex, Garuda International, Aloha Medicinals, Huisong Pharmaceuticals, Zhejiang Fangge Pharmaceutical Co., Ltd., and Fungi Health.

**Table 1:** Commercially available mushroom preparations used in this study.

Source	Mushroom part	Ref ID	Drug-extract ratio
Reishi	Fruiting body, mycelium, growth medium	1622	WM
Reishi	Fruiting body	1628	4:1
Reishi	Fruiting body, mycelium, growth medium	1639	WM
Reishi	Fruiting body, mycelium	1631	66:1
Shiitake	Fruiting body, mycelium, growth medium	1634	WM
Shiitake	Fruiting body	1630	4:1
Shiitake	Fruiting body	1633	4:1
Maitake	Fruiting body	1629	4:1
Maitake	Fruiting body, mycelium, growth medium	1635	WM
Reishi	Fruiting body	M18–1	16:1
Maitake	Fruiting body	M18–2	4:1
Shiitake	Fruiting body	M18–3	4:1
Formula:	Fruiting bodies	M18–13	
Reishi		M18–1	16:1 (10 parts)
Maitake		M18–2	4:1 (33 parts)
Shiitake		M18–3	4:1 (40 parts)

WM: Whole mushroom

### Quantification of α- and β-glucans in mushroom extracts

α- and β-Glucan content was measured using the Megazyme kit K-YBGL (Megazyme Inc. IL. USA), essentially as previously described [10].

#### Measurement of α-glucan (starch/glycogen)

Approximately 100 mg (weighed accurately) of the sample was added to a 20 × 125 mm screw capped tube, and the tube was tapped to ensure that the entire sample fell to the bottom of the tube. A magnetic stirrer bar and 2.0 mL of ice-cold 2 M KOH was added to each tube, and the tube contents were stirred using a magnetic stirrer in an ice water bath for 20 min to dissolve the starch/glycogen. 1.2 M sodium acetate buffer (pH 3.8; 8 mL) was added to each tube with mixing on a vortex stirrer. Amyloglucosidase (1630 U/mL) plus invertase (500 U/mL) (200μL) (from Megazyme kit) was immediately added, the contents were mixed well, and the tubes were incubated at 40°C for 30 min. This solution (10.3 mL final volume) was centrifuged at 1500 rpm for 10 min and 0.1 mL of the supernatant solutions was analysed for glucose with glucose oxidase/peroxidase reagent.

#### Measurement of total glucan

Approximately 100 mg (weighed accurately) of the sample was added to a 20 × 125 mm screw capped tube, and the tube was tapped to ensure that the entire sample fell to the bottom of the tube. A total of 2.0 mL of ice-cold 12 M sulfuric acid was added to each tube, and the tubes were capped and stirred on a vortex mixer. Tubes were placed in an ice–water bath and left for 2 h. During this time, the tube’s contents were vigorously stirred (for 10–15 s) several times on a vortex mixer to ensure complete dissolution/dispersion of the sample. Water (2 × 5mL) was added in two portions to each tube, and the tubes were capped and vigorously stirred on a vortex mixer for 10 s. The caps were loosened and the tubes were placed in a hot-block heater (~100°C). After 5 min, the caps were tightened and the incubation was continued at 100°C for 2 h. The tubes were cooled to room temperature, and the caps were carefully loosened. 10 M KOH (6 mL) was added, and the tube contents were mixed well. The contents of each tube were quantitatively transferred to 100 mL volumetric flasks using 200 mM sodium acetate buffer (pH 5), and the volume was adjusted to 100 mL with 200 mM sodium acetate buffer (pH 5). The contents were mixed thoroughly, and an aliquot (~10 mL) of the solution was centrifuged 1500rpm for 10 min in a bench centrifuge. 100 μL of the sample solution was incubated with 100 μL of a mixture of *exo*-1,3-β-glucanase (20 U/mL) plus β-glucosidase (4 U/mL) at 40°C for 60 min, and the glucose was determined with GOPOD reagent as previously described (all of the reagents used were in the Megazyme kit). Absorbance was measured at 510 nm. Concurrently, a 0.1 mL aliquot of glucose standard solution (1 mg/mL), was incubated in quadruplicate (standard) with GOPOD reagent; also, 0.1 mL of acetate buffer (200 mM, pH 5) was incubated with 3.0 mL of GOPOD reagent (reagent blank).

#### Determination of β-glucan

The β-glucan content was determined by subtracting the α-glucan content from the total glucan content.

### Macrophage cell model

Human peripheral blood was obtained from a healthy human volunteer. The participant received a written information sheet, and consent was obtained with a signed consent form. The collection of human peripheral blood as outlined in Griffith University Human Research Ethics Committee reference number 2016/713 was approved by the Griffith University Human Research Ethics Committee. Isolation and differentiation of human macrophages was performed using an adaptation of a previously described protocol [13]. Briefly, healthy human peripheral blood was collected in lithium heparin vacuettes (Wishmed, NSW, AUS) and inverted 5 times to prevent coagulation. Initially 15 mL of Histopaque^®^-1077 (Sigma-Aldrich Co. LLC, MO, USA) solution at room temperature was added into two 50 mL centrifuge tubes and 25 mL of peripheral blood was layered on top carefully without disturbing the Histopaque layer. Both tubes were centrifuged at 400*g* without brake for 30 minutes at room temperature to generate a density gradient. First the layer of plasma was aspirated out and discarded, subsequently the layer of PBMC as far into the Histopaque layer without disturbing the red blood cells or neutrophils was collected and placed into a fresh tube. The PBMCs were then washed with RPMI 1640 medium (Thermo Fisher Scientific, MA, USA) in a 1:1 solution and centrifuged for 10 minutes at 300*g*, 10°C with the brake on. The resulting supernatant was discarded and the RPMI wash step repeated with 10 mL of RMPI under the same conditions. Again, supernatant was discarded and the pellet resuspended in culture medium consisting of RPMI with 1% penicillin/streptomycin, 2.5 ng/mL M-CSF (macrophage colony-stimulating factor, Thermo Fisher Scientific, MA, USA) and 10% NBCS (Newborn Calf Serum, Thermo Fisher Scientific, MA, USA). Cells were incubated in Greiner 24 Well TC Plate (Interpath Services, VIC, AUS) for 14 days at 37°C with 5% CO_2_ at a density of 2x10^5^ cells/mL to allow the cells to adhere prior to any experiments being conducted. The behaviour and morphology of human macrophages generated in this way were consistent with that previously published [13].

### Cytokine expression analysis

Cells were treated with fresh media containing varying concentration of mushroom extracts or formula (1, 10, 100, 1,000 μg/mL) with or without the addition of 1 μg/mL of LPS (Sigma-Aldrich Co. LLC, MO, USA) and incubated for an additional 72 hours (24 hours for determination of TNF-α). Subsequently cell culture supernatant was collected and stored at -80°C. The cytokine level present in this supernatant was determined using ELISA-based assays supplied by elisakit.com (VIC, AUS), with the concentration of cytokine present in the cell culture supernatant (samples and controls) determined using a standard curve as described by the manufacturer.

### Statistical analysis

Data generated from the ELISA assays were used to produce a Nonlinear Regression Curve performed using GraphPad Prism version 7.03 (Windows, GraphPad Software, CA, USA) to determine the EC_50_/IC_50_ values of the three mushroom extracts and mushroom formula. The parameters of the slope utilised was a variable slope with least squares (ordinary) fit and interpolate unknowns from a standard curve, generated with the following equation

Y=Bottom+(Top−Bottom)/(1+10^((LogEC50−X)*HillSlope)).

A response additivity plot was generated to determine the additive effect of the mushroom formula. The additive line was obtained by summing the cytokine expression levels that were proportional to the percentage of extracts used in the mushroom formula. A response above this additive line indicates a synergistic effect between the extracts and beneath the line an antagonistic effect [14]. Another dose–effect curve approach, curve-shift analysis, was also generated from the additive effect of the extracts on the individual dose–effect curves, an example of two extracts can be expressed using the equation, Effect(x+y) = E_X_(x+x_y_) = E_Y_(y_x_+y) = E_XY_, where E_X_ is the sum of the dose-effect from extract x plus the effect of extract y at the same dose as x. This curve allows for identifying synergy in two ways, increase in potency and/or an increase in efficacy relatively to the additive extract response [14].

## Results and discussion

The determination of α- and β-glucan content in commercially available mushroom extracts using the Megazyme method [10] found that six of the mushroom powders contain 10–20% α-glucan (Table 2).

**Table 2:** β-and α-Glucan composition of commercially available mushroom preparations used in this study.

Source	Ref ID	β-glucan (%)	α-glucan (%)	Total glucan (%)	Ratio of β-:α-glucan	Ratio of β-:total glucan
Reishi	1622	38.6	12.6	51.2	3.1	0.75
Reishi	1628	38.0	1.1	39.1	34.5	0.97
Reishi	1639	31.0	17.8	48.8	1.7	0.64
Reishi	1631	21.4	13.9	35.3	1.5	0.61
Shiitake	1634	36.7	15.3	52.0	2.4	0.71
Shiitake	1630	12.3	1.2	13.5	10.0	0.91
Shiitake	1633	10.2	0.8	10.9	13.6	0.93
Maitake	1629	18.6	1.3	19.9	14.9	0.94
Maitake	1635	40.6	13.3	53.9	3.1	0.75
Reishi	M18–1	17.7	3.2	20.9	5.5	0.85
Maitake	M18–2	32.0	3.8	35.8	8.4	0.89
Shiitake	M18–3	20.1	0.8	20.9	25.1	0.96
Formula	M18–13	22.1	3.2	25.3	6.9	0.87

This high level of α-glucan is not naturally found in mushrooms [5] and likely indicates the presence of non-mushroom glucans, such as those found in growth media (e.g. brown rice, potato starch) and/or excipients (e.g. maltodextrin). As such the presence of glucans from extraneous sources changes the overall glucan profile as revealed by the changes in β-glucan:total glucan ratio (Table 2). The growth medium, for example, may contain β-glucan as well as α-glucan (e.g. potato starch), resulting in relatively high levels of α-glucan as well as an increase in β-glucan that is not derived from the mushroom. This means that it is not possible to compare mushroom fruiting body products with mycelium-containing products on the basis of the β-glucan content alone. More importantly, traditional use of these mushrooms was based solely on the fruiting body, and it is a more recent innovation, through the advent of bio-fermentation technologies, that full spectrum products are now appearing in the market. Naturally, the presence of glucans from non-mushroom sources means the purity of the product may be compromised. Based on the data shown in Tables 1 and 2 we generated a formula comprising Reishi, Shiitake and Maitake fruiting body extracts with the high β-glucan:α-glucan and β-glucan:total glucan ratios, with a view to preparing a formula that more effectively expressed the immunomodulating activities reported with mushroom β-glucan. The composition of this formula was Reishi fruiting body extract (M18–1, 10 parts), Shiitake fruiting body extract (M18–3, 40 parts) and Maitake fruiting body extract (M18–2, 33 parts), giving the Maitake:Reishi:Shiitake ratio as 1.0:1.2:1.2 on a dry weight equivalent basis (Research from this study has resulted in the development of a product released by Integria Healthcare. This formula is marketed in Australia and New Zealand as MediHerb^®^ Mushroom Forte (AUSTL301609)). This combination was found to have a β-glucan:α-glucan ratio of 6.9 and a β-glucan:total glucan ratio of 0.87. It has been reported that macrophages express cytokines in response to stimulation by medicinal mushrooms [15]. We therefore assessed the ability of our mushroom formula, and the individual extracts that comprise the formula, to impact the expression of IL-1α, IL-6, TNF-α and IL-10 in human macrophages with and without LPS stimulation by determining EC_50_ or IC_50_ values. The cytokines evaluated were chosen to cover both pro- and anti-inflammatory cytokines and included those commonly used to monitor the capacity of natural product preparations to elicit an immune response [16]. In addition to the standard pro-inflammatory cytokines IL-6 and TNF-α, we also monitored the expression of IL-1α over the more commonly evaluated IL-1β, as expression of IL-1α is known to induces tumour regression [17].

In a similar way to that observed by others [18–21], the majority of individual mushroom extracts induced dose-dependent increases in cytokine expression (S1–S4 Figs). Likewise, the formula (M18–13) induced dose-dependent increases in cytokine expression in both non-LPS and LPS treated macrophages (S1 Fig), indicating that the mushroom formula was potentially acting as an immunostimulant. To further explore the immunostimulatory effects of the mushroom formula and its component extracts, EC_50_ (concentration that gives half-maximal response) and IC_50_ (concentration that inhibits response by half) values were determined by a nonlinear regression variable slope. The majority of the EC_50_ values were <100 μg/mL (Table 3), with the mushroom formula having EC_50_ values lower in the LPS stimulated macrophages compared to non-stimulated counterparts. Interestingly and unexpectedly, the EC_50_ values for the mushroom formula on LPS stimulated macrophages were lower in comparison to the individual mushroom extracts that comprise the formula for TNF-α, and second lowest for IL-6 and IL-10 (Table 3). These observations suggested a potential synergistic effect between the individual components that make up the mushroom formula. As such further analyses were performed to explore this significant phenomenon in more detail.

**Table 3:** EC_50_ and IC_50_ values associated with the effect of mushroom extracts and formula on cytokine expression in non-LPS (−) and LPS (+) stimulated human macrophages.

	EC_50_ or IC_50_ (μg/mL)							
	Shiitake (M18–3)		Reishi (M18–1)		Maitake (M18–2)		Formula (M18–13)	
	−LPS	+LPS	−LPS	+LPS	−LPS	+LPS	−LPS	+LPS
TNF-α	48.7	19.4	58.6	70.9	65.1	73.0	45.4	7.1
IL-1α	7.0	82.8	778.4	N.D.	14.4*	5.4*	434.1	177.7
IL-6	29.1	43.2	49.8	71.0	75.8	7.0	45.8	22.5
IL-10	20.1	34.9	31.0	78.0	5.1	6.3	25.6	16.0

Data derived from a nonlinear regression variable slope with interpolated unknowns from standard curve (S5–S8 Figs), EC_50_ determined from sample concentrations. N.D. Not Determined as slope was unable to converge.

*Indicates IC_50_ values.

**Fig 1:**
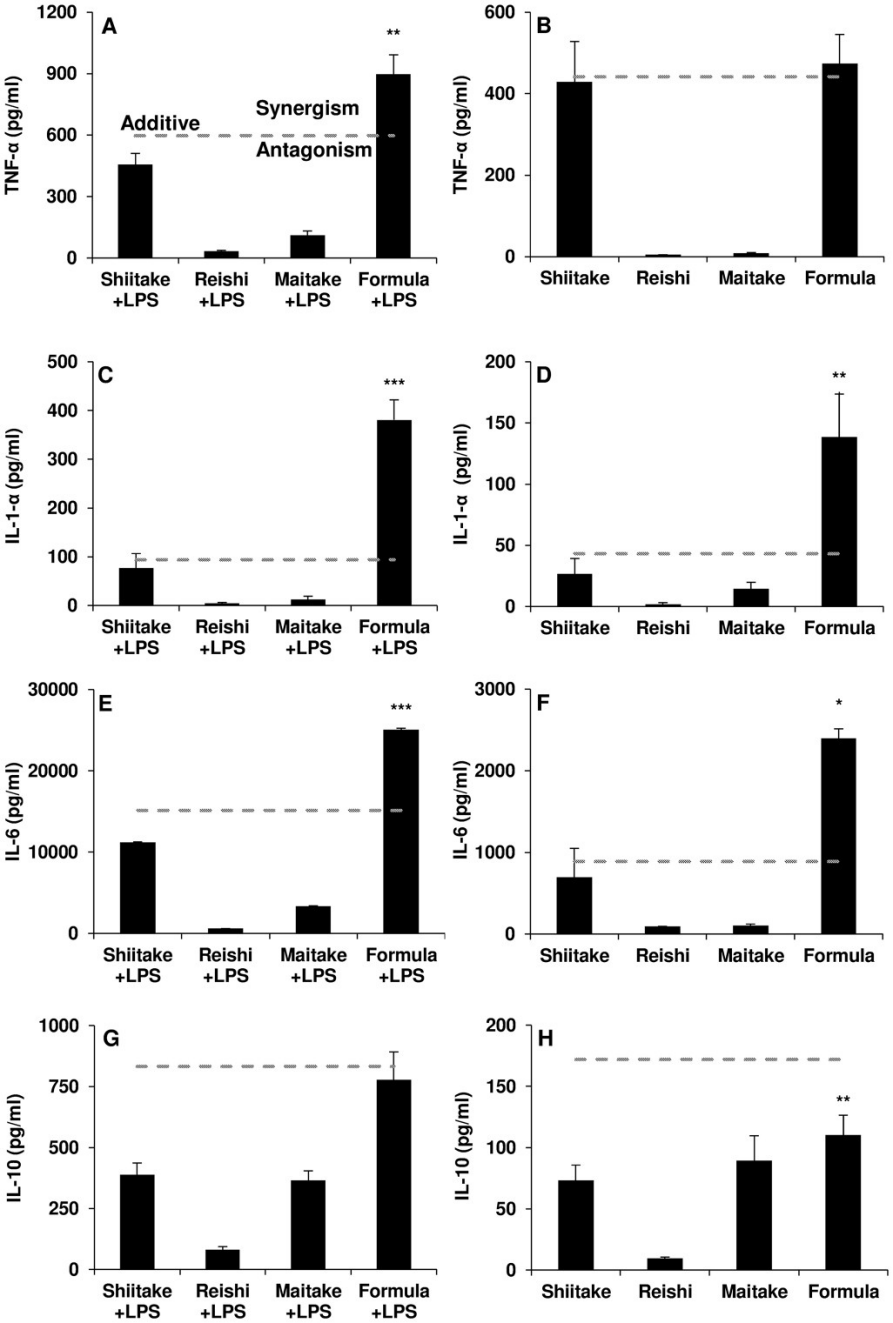
Effect-based response additivity for cytokine expression induced by mushroom extracts and formula in LPS stimulated and non-stimulated human macrophages. The expected additive effect (dashed line) represents the level of expression expected if the response derived from the mushroom formula (M18–13) was simply the sum of the individual responses of its components (M18–1, M18–2, M18–3). A response above the additive line indicates a potential synergistic effect between the extracts and beneath the line a potential antagonistic effect. (A) TNF-α, (C) IL-1α, (E) IL-6 and (G) IL10 expression in LPS stimulated macrophages; (B) TNF-α, (D) IL-1α, (F) IL-6 and (H) IL-10 expression in non-LPS stimulated macrophages. Mushroom preparation concentration 100 μg/mL. Results shown as mean + SEM, statistics are Student’s *t*-test *P < 0.07, **P < 0.05 and ***P < 0.01.

To determine the effect-based responses derived by the mushroom formula, a response additivity graph was constructed for each cytokine investigated (Fig 1A–1H). This illustrates the proportional effect on cytokine expression of the individual extracts comprising the mushroom formula, in addition to the combination effect of the formula itself. Summing the effects of the individual extracts generates the additive line. This line represents the level of expression expected if the response derived from the mushroom formula was simply the sum of the individual proportional responses of its components. However, the response additivity graph only reflects a drug’s efficacy, not its potency. As seen in Fig 1A, TNF-α expression for the mushroom formula in LPS stimulated macrophages was significantly greater (P < 0.05) than that of the additive line, indicating a potential synergistic effect. This statistically significant synergistic effect was also observed for the majority of cytokines in both LPS stimulated and non-LPS human macrophages (Fig 1A, 1C–1F), except for TNF-α in non-stimulated macrophages and IL-10 in both LPS stimulated and non-LPS human macrophages (Fig 1B, 1G and 1H). Interestingly, IL-10 showed a potential antagonistic effect where the response as a result of the mushroom formula was less than the summed responses of the individual extracts (Fig 1G and 1H), with statistical significance observed in non-stimulated macrophages (Fig 1H). It should be noted that IL-10 expression as a result of treatment with the mushroom formula was still greater than the IL-10 expression observed in the LPS stimulated macrophage control. Taken together, our data suggests that the mushroom formula is inducing an immunostimulatory response in a synergistic manner, by inducing the expression the pro-inflammatory IL-1α, IL-6 and TNF-α, and reducing the expression of the anti-inflammatory IL-10 (compared to the summed responses of the individual extracts).

**Fig 2:**
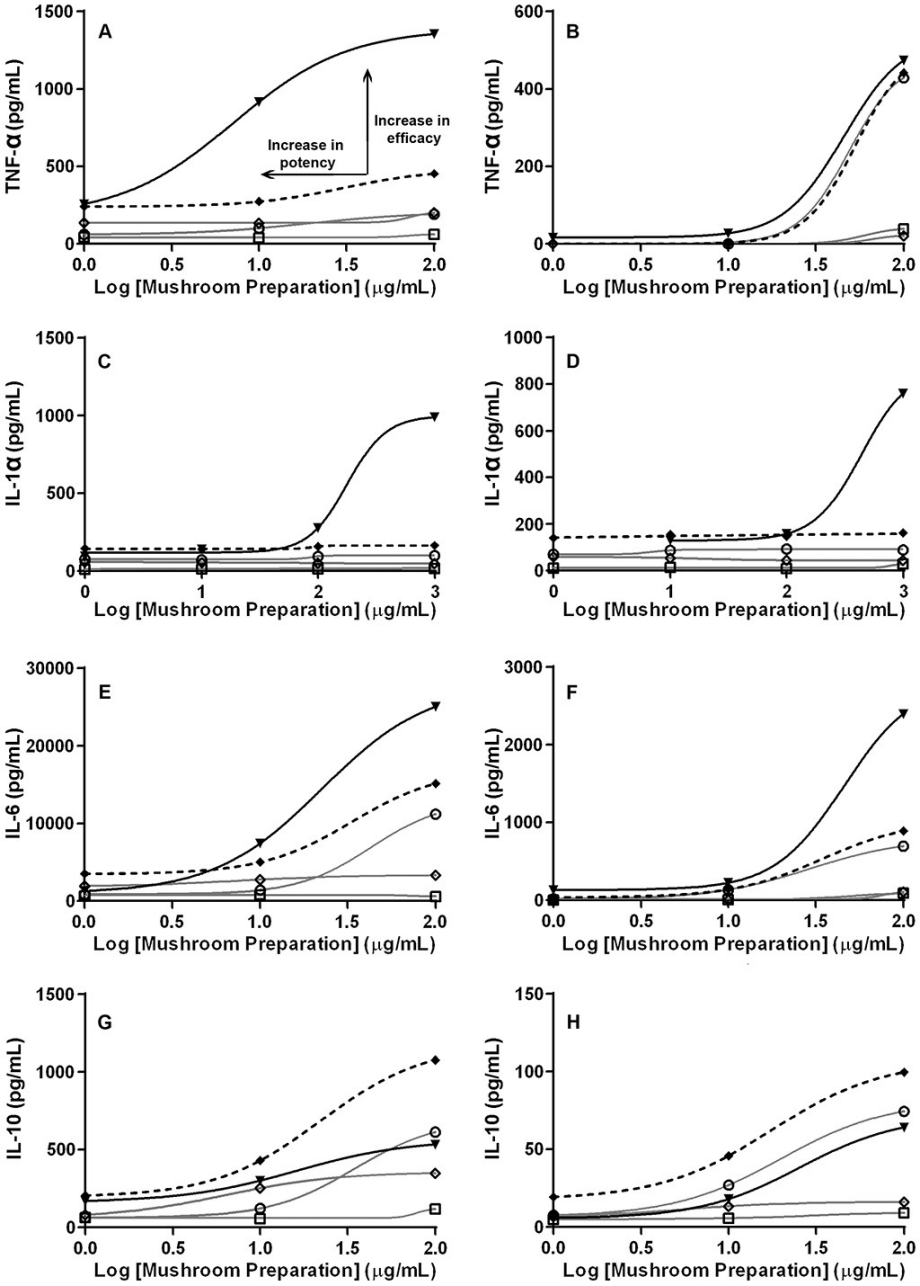
Curve-shift analysis for cytokine expression induced by mushroom extracts and formula in LPS stimulated and non-stimulated human macrophages. The dose–effect curve for mushroom formula (M18–13) (▼ solid black line) compared to the expected additive effect of the individual extract responses (◆ dashed black line), and the individual mushroom extracts Shiitake (M18–2) (⚪ grey line), Reishi (M18–1) (□ grey line) and Maitake (M18–3) (◇ grey line). (A) TNF-α, (C) IL-1α, (E) IL-6 and (G) IL10 expression in LPS stimulated macrophages; (B) TNF-α, (D) IL-1α, (F) IL-6 and (H) IL-10 expression in non-LPS stimulated macrophages. Curve-shift analysis allows synergistic effect to be measured in two separate ways, as an increase in potency and/or efficacy indicated by an increase in the slope of the curve relative to the combined effect of the individual extract responses.

A known limitation inherent to the response additivity design is the assumption that the dose-response curves are linear with a zero intercept [14]. As such a curve-shift analysis was subsequently performed (Fig 2), which allowed us to measure and confirm the putative synergistic effect in two separate ways: an increase in potency and/or efficacy, indicated by a curve-shift to the left and an increase in the slope of the curve relative to the combined effect of the individual extract responses, respectively. As seen in Fig 2A for TNF-α, the mushroom formula (solid black line) demonstrated an increase in both potency and efficacy over the additive effects of the extracts (dashed black line), indicating that the mushroom formula evoked a greater response at lower concentration as well as inducing an overall larger cytokine expression response than the sum effect produced by the individual extracts. Apart from IL-10, the remaining curve-shift analyses (Fig 2) illustrated the formula’s synergistic effect with an increase in efficacy for TNF-α, IL-6 and IL-1α. As well as being more potent at all concentrations for TNF-α and IL-6 for non LPS treated macrophages (Fig 2A, 2B and 2F), increased potency was also noted at higher concentrations (≥100 μg/mL) for IL-1α and IL-6 for LPS treated macrophages (Fig 2C, 2D and 2E). Interestingly, the formula had an antagonistic effect on IL-10 cytokine expression in both non and LPS stimulated macrophages, with both potency and efficacy being lower compared with the additive effects of the three extracts (Fig 2G and 2H).

The observed synergistic induction of pro-inflammatory cytokines together with the antagonistic effect on IL-10 by our mushroom formula could be explained by considering the interplay between pro- and anti-inflammatory cytokine expression during the innate immune response. Macrophages are a main component of the innate immune system, this system acts as the body’s first line of defence against infection [5]. The innate immune system utilises pattern recognition receptors (PRRs) that recognise pathogen-associated molecular patterns (PAMPs) to induce cytokine production within macrophages. The activation of macrophages induces the secretion of proinflammatory cytokines such as TNF-α and IL-6, which are followed by the secretion of anti-inflammatory cytokines. The pro-inflammatory cytokines have protective roles in acute inflammatory responses, however an overexpression of these cytokines can lead to chronic diseases [22–24]. The temporal release of anti-inflammatory cytokine IL-10 is indicative of a feedback control mechanism that is used to prevent dysregulation of the pro-inflammatory cytokines [24, 25]. Studies have shown that the addition of IL-6 to alternatively activated macrophages induced IL-10 expression [5], whereas in PBMC co-stimulation with both TNF-α and Bt2cAMP also induced the expression of IL-10 [25].

As previously stated, to develop a formula that can effectively stimulate immunomodulating activities, a mushroom formula was prepared using a combination of mushroom extracts with high β-glucan:total glucan ratios. In order to further explore any potential correlation between glucan content and immunomodulatory activity, coefficient of determination statistics were calculated for cytokine expression levels versus percentage β-glucan, α-glucan, total glucan, and β-glucan:total glucan ratio. Correlation (R^2^) statistics for the expression of IL-1α, IL-10 and TNF-α in both LPS stimulated and non-stimulated macrophages following exposure to the mushroom extracts listed in Table 1 showed that the strongest positive correlation was between β-glucan:total glucan ratio and IL-1α expression (R^2^ = 0.156) (S1 Table and S9–S12 Figs). This suggests that the increased expression of IL-1α in non-stimulated macrophages (S1 Table) may be correlated with the β-glucan:total glucan ratio. This is a particularly interesting finding given that the expression of IL-1α is known to induces tumour regression [17]. Interestingly the largest negative correlation was also observed between IL-1α in non-stimulated macrophages and percentage α-glucan content (R^2^ = 0.229) present in the extract.

However, clearly the overall correlation strength is weak, indicating that the observed immunomodulatory activity is not derived solely from the quantity of β-glucan or α-glucan, but rather may be due to the nature of the β-glucan structures present. It is therefore critical that future work explores the exact nature of the β-glucan structures that elicit specific immune responses.

## Conclusions

The concept of synergy is well established in the natural products field, with for example synergy reported between plant natural products for the prevention and/or treatment of cancer [26, 27] and malaria [28], and between natural products and antibiotics for the treatment of infectious disease [29]. Even though synergistic immunomodulatory effects between plant natural products have been reported [30, 31], to our knowledge this is the first report of synergistic immunomodulatory activity between defined mushroom preparations. Specifically, this study revealed the first observation of a synergistic effect of a mushroom formula on the expression of the IL1-α, IL-6 and TNF-α, and an antagonistic effect on the expression of IL-10. Further studies are required to more fully explore the synergistic immunomodulatory effect observed by our mushroom formula, particularly determining the affinity and extent of response induced by the interaction of well-defined, structurally elucidated mushroom β-glucans with recognised pathogen-associated molecular patterns (PAMPs), and the pattern recognition receptors (PRRs) present on macrophages.

## Supporting information

S1 Fig Triplicate repeats of the effect of a mushroom formula on cytokine expression on LPS stimulated and non-stimulated human macrophages. Concentrations are represented by log value and range from 1 to 1,000 μg/ml. Formula on LPS stimulated macrophages (black solid line), Formula on non-LPS stimulated macrophages (dashed line), Control (no formula) on LPS stimulated macrophages (horizontal dashed line), Control (no formula) on LPS stimulated macrophages (dotted line). (A) TNF-α, (B) IL-1α, (C) IL-6, (D) IL-10.
(TIF)

S2 Fig Triplicate repeats of Shiitake the effect of a on cytokine expression on LPS stimulated and non-stimulated human macrophages. Concentrations are represented by log value and range from 1 to 1,000 μg/ml. Shiitake on LPS stimulated macrophages (black solid line), Shiitake on non-LPS stimulated macrophages (dashed line), Control (no Shiitake) on LPS stimulated macrophages (horizontal dashed line), Control (no Shiitake) on LPS stimulated macrophages (dotted line). (A) TNF-α, (B) IL-1α, (C) IL-6, (D) IL-10.
(TIF)

S3 Fig Staggered Triplicate repeats on the effect of cytokine expression by varying Reishi concentrations compared to control cytokine expression on ± LPS stimulated human macrophages. Concentrations are represented by log value and range from 1 to 1,000 μg/ml. Reishi on LPS stimulated macrophages (black solid line), Reishi on non-LPS stimulated macrophages (dashed line), Control (no Reishi) on LPS stimulated macrophages (horizontal dashed line), Control (no Reishi) on LPS stimulated macrophages (dotted line). (A) TNF-α, (B) IL-1α, (C) IL-6, (D) IL-10.
(TIF)

S4 Fig Staggered Triplicate repeats on the effect of cytokine expression by varying Maitake concentrations compared to control cytokine expression on ± LPS stimulated human macrophages. Concentrations are represented by log value and range from 1 to 1,000 μg/ml. Maitake on LPS stimulated macrophages (black solid line), Maitake on non-LPS stimulated macrophages (dashed line), Control (no Maitake) on LPS stimulated macrophages (horizontal dashed line), Control (no Maitake) on LPS stimulated macrophages (dotted line). (A) TNF-α, (B) IL-1α, (C) IL-6, (D) IL-10.
(TIF)

S5 Fig Nonlinear regression EC_50_ curve of mushroom formula effect on cytokine expression in ± LPS stimulated human macrophages. Concentrations are represented by log value and range from 1 to 1,000 μg/ml. Formula on LPS stimulated macrophages (black solid line), Formula on non-LPS stimulated macrophages (dashed line). (A) TNF-α, (B) IL-1α, (C) IL-6, (D) IL-10.
(TIF)

S6 Fig Nonlinear regression EC_50_ curve of Shiitake effect on cytokine expression in ± LPS stimulated human macrophages. Concentrations are represented by log value and range from 1 to 1,000 μg/ml. Shiitake on LPS stimulated macrophages (black solid line), Shiitake on non-LPS stimulated macrophages (dashed line). (A) TNF-α, (B) IL-1α, (C) IL-6, (D) IL-10.
(TIF)

S7 Fig Nonlinear regression EC_50_ curve of Reishi effect on cytokine expression in ± LPS stimulated human macrophages. Concentrations are represented by log value and range from 1 to 1,000 μg/ml. Reishi on LPS stimulated macrophages (black solid line), Reishi on non-LPS stimulated macrophages (dashed line). (A) TNF-α, (B) IL-1α, (C) IL-6, (D) IL-10.
(TIF)

S8 Fig Nonlinear regression EC_50_ curve of Maitake effect on cytokine expression in ± LPS stimulated human macrophages. Concentrations are represented by log value and range from 1 to 1,000 μg/ml. Maitake on LPS stimulated macrophages (black solid line), Maitake on non-LPS stimulated macrophages (dashed line). (A) TNF-α, (B) IL-1α, (C) IL-6, (D) IL-10.
(TIF)

S9 Fig Coefficient of determination statistics calculated for IL-1α in LPS non-stimulated macrophages. Expression levels versus percentage β-glucan (a), α-glucan (b), total glucan (c), β-glucan: total glucan ratio (d) on LPS non-stimulated macrophages following exposure to the mushroom extracts listed in Table 1.
(TIF)

S10 Fig Coefficient of determination statistics calculated for IL-1α in LPS stimulated macrophages. Expression levels versus percentage β-glucan (a), α-glucan (b), total glucan (c), β- glucan: total glucan ratio (d) on LPS stimulated macrophages following exposure to the mushroom extracts listed in Table 1.
(TIF)

S11 Fig Coefficient of determination statistics calculated for IL-10 in LPS non-stimulated macrophages. Expression levels versus percentage β-glucan (a), α-glucan (b), total glucan (c), β-glucan: total glucan ratio (d) on LPS non-stimulated macrophages following exposure to the mushroom extracts listed in Table 1.
(TIF)

S12 Fig Coefficient of determination statistics calculated for IL-10 in LPS stimulated macrophages. Expression levels versus percentage β-glucan (a), α-glucan (b), total glucan (c), β-glucan: total glucan ratio (d) on LPS stimulated macrophages following exposure to the mushroom extracts listed in Table 1.
(TIF)

S13 Fig Coefficient of determination statistics calculated for TNF-α in LPS non-stimulated macrophages. Expression levels versus percentage β-glucan (a), α-glucan (b), total glucan (c), β-glucan: total glucan ratio (d) on LPS non-stimulated macrophages following exposure to the mushroom extracts listed in Table 1.
(TIF)

S14 Fig Coefficient of determination statistics calculated for TNF-α in LPS stimulated macrophages. Expression levels versus percentage β-glucan (a), α-glucan (b), total glucan (c), β-glucan: total glucan ratio (d) on LPS stimulated macrophages following exposure to the mushroom extracts listed in Table 1.
(TIF)

S1 Data Excel file containing raw data associated with figures (including supporting information figures) presented in this study. (XLS)

S1 Table Summary of coefficient of determination. R^2^ values for β-glucan, α-glucan, total glucan, and β-glucan: total glucan composition data between the mushroom preparations listed in Table 1 and cytokine expression in human macrophages ± LPS stimulation.
(TIF)
