# Supplementary figures and images for: Synergistic immuno-modulatory activity in human macrophages of a medicinal mushroom formulation consisting of Reishi, Shiitake and Maitake
Source: PLoS One. 2019 Nov 7;14(11):e0224740. doi: 10.1371/journal.pone.0224740 (PMC6837746; doi:10.1371/journal.pone.0224740)

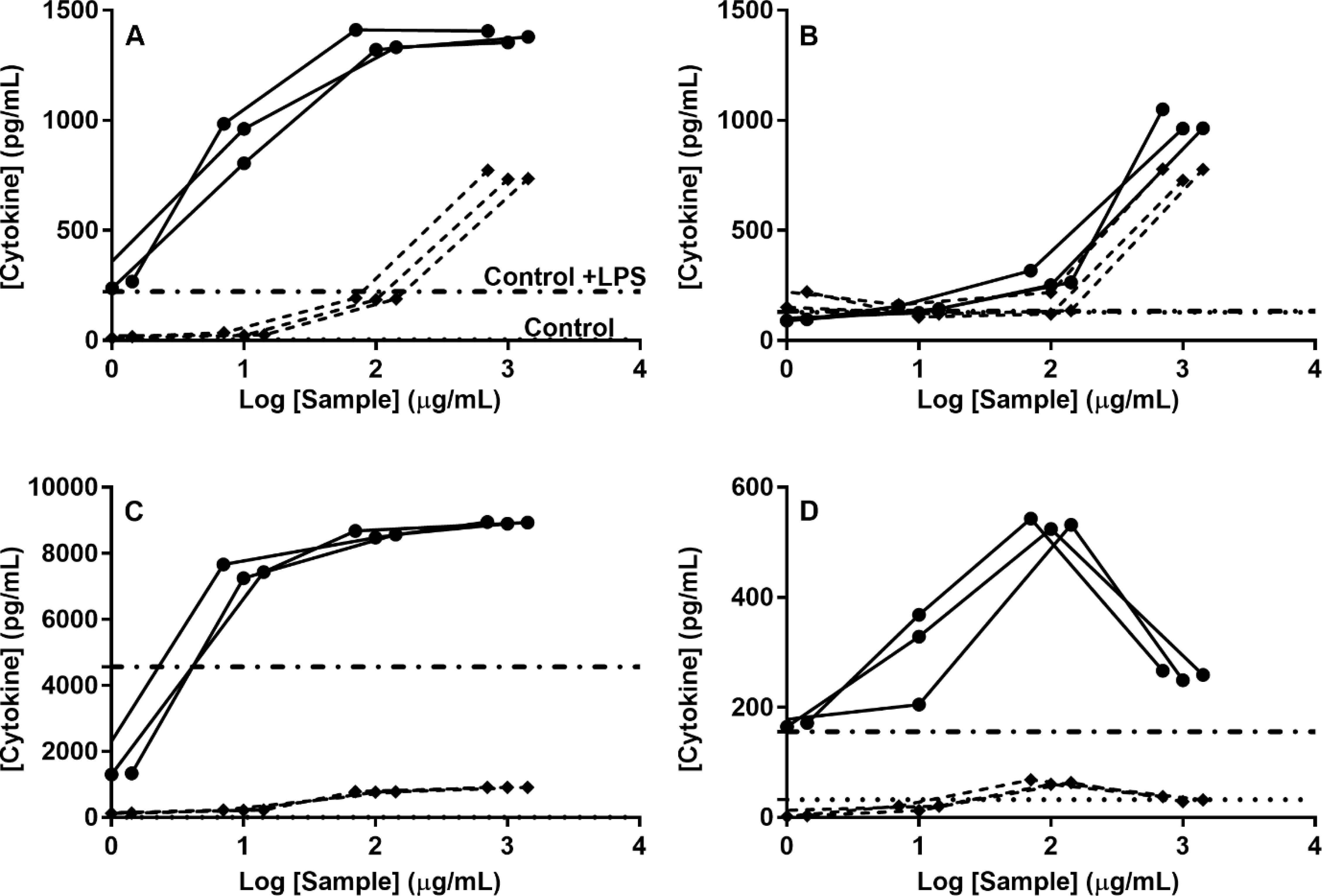

Supplement: S1 Fig — Concentrations are represented by log value and range from 1 to 1,000 μg/ml. Formula on LPS stimulated macrophages (black solid line), Formula on non-LPS stimulated macrophages (dashed line), Control (no formula) on LPS stimulated macrophages (horizontal dashed line), Control (no formula) on LPS stimulated macrophages (dotted line). (A) TNF-α, (B) IL-1α, (C) IL-6, (D) IL-10. (TIF) [file pone.0224740.s001.tif]

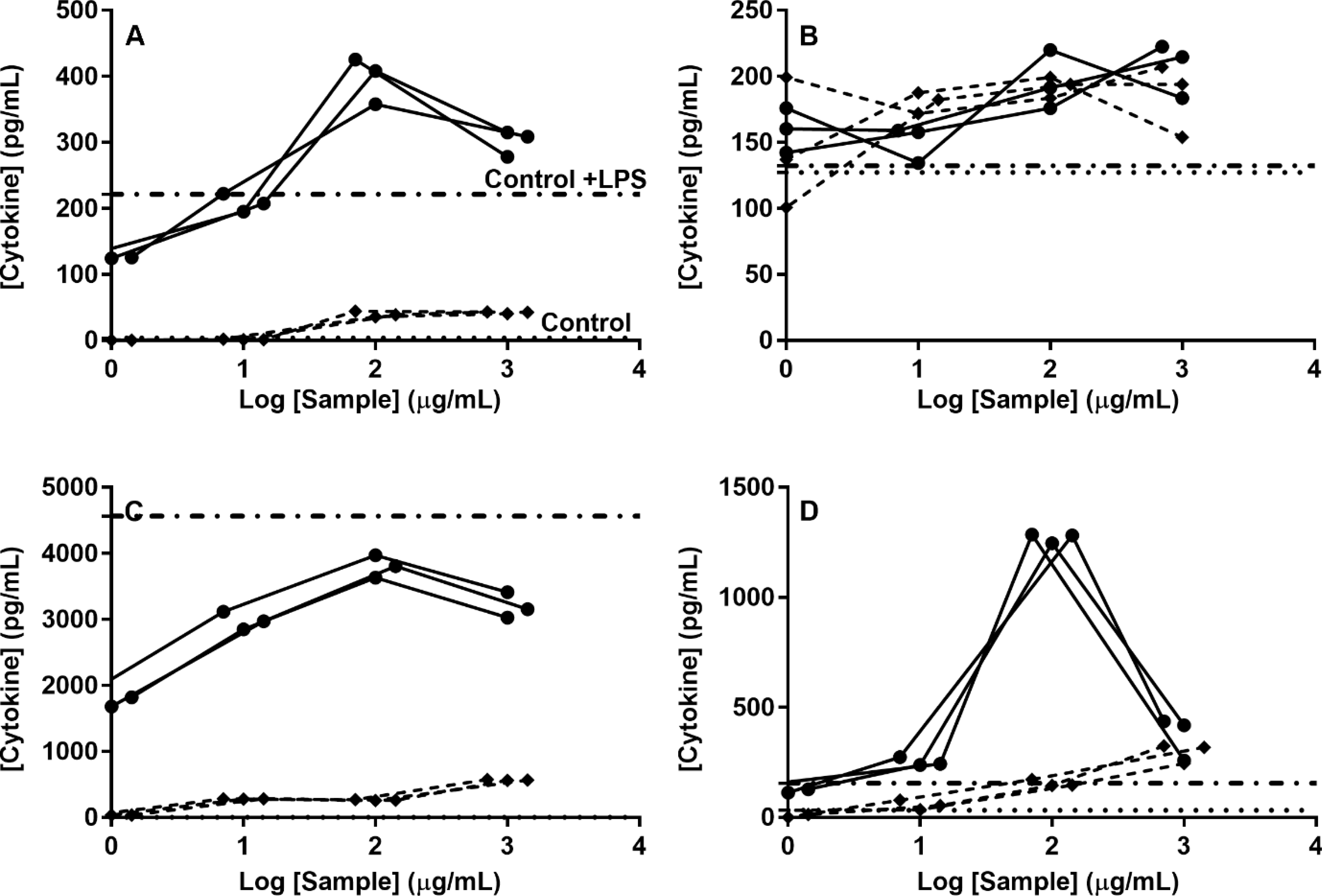

Supplement: S2 Fig — Concentrations are represented by log value and range from 1 to 1,000 μg/ml. Shiitake on LPS stimulated macrophages (black solid line), Shiitake on non-LPS stimulated macrophages (dashed line), Control (no Shiitake) on LPS stimulated macrophages (horizontal dashed line), Control (no Shiitake) on LPS stimulated macrophages (dotted line). (A) TNF-α, (B) IL-1α, (C) IL-6, (D) IL-10. (TIF) [file pone.0224740.s002.tif]

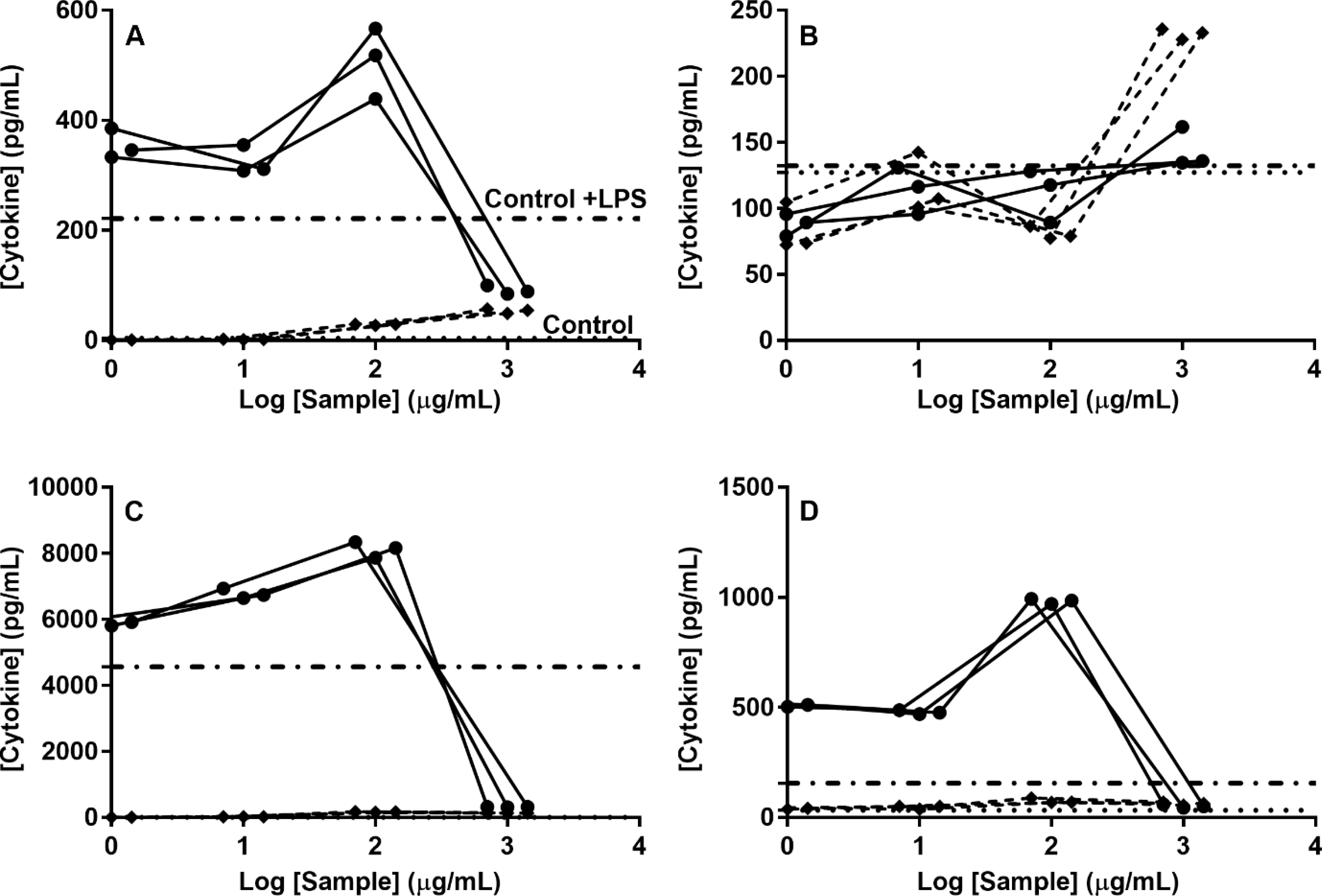

Supplement: S3 Fig — Concentrations are represented by log value and range from 1 to 1,000 μg/ml. Reishi on LPS stimulated macrophages (black solid line), Reishi on non-LPS stimulated macrophages (dashed line), Control (no Reishi) on LPS stimulated macrophages (horizontal dashed line), Control (no Reishi) on LPS stimulated macrophages (dotted line). (A) TNF-α, (B) IL-1α, (C) IL-6, (D) IL-10. (TIF) [file pone.0224740.s003.tif]

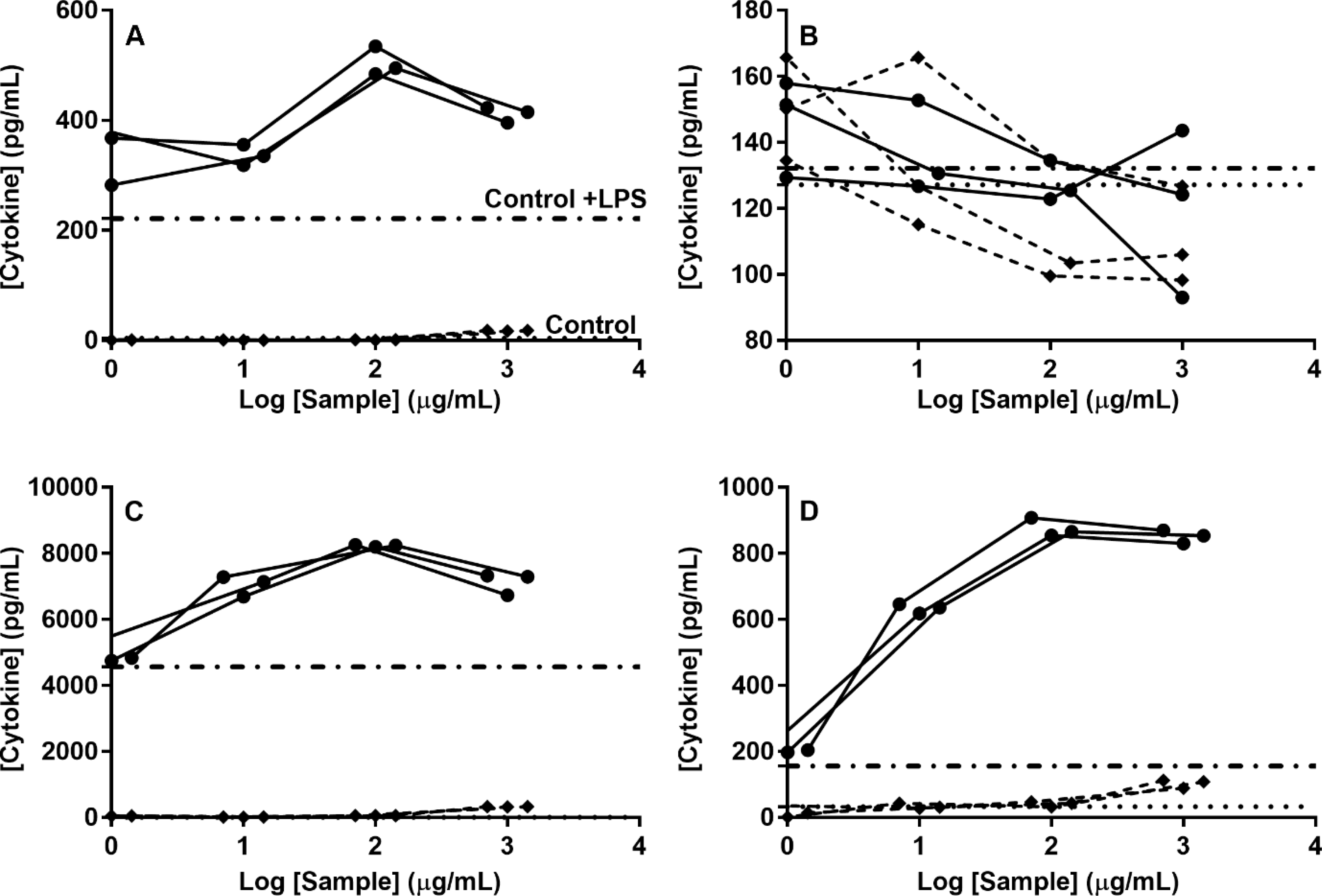

Supplement: S4 Fig — Concentrations are represented by log value and range from 1 to 1,000 μg/ml. Maitake on LPS stimulated macrophages (black solid line), Maitake on non-LPS stimulated macrophages (dashed line), Control (no Maitake) on LPS stimulated macrophages (horizontal dashed line), Control (no Maitake) on LPS stimulated macrophages (dotted line). (A) TNF-α, (B) IL-1α, (C) IL-6, (D) IL-10. (TIF) [file pone.0224740.s004.tif]

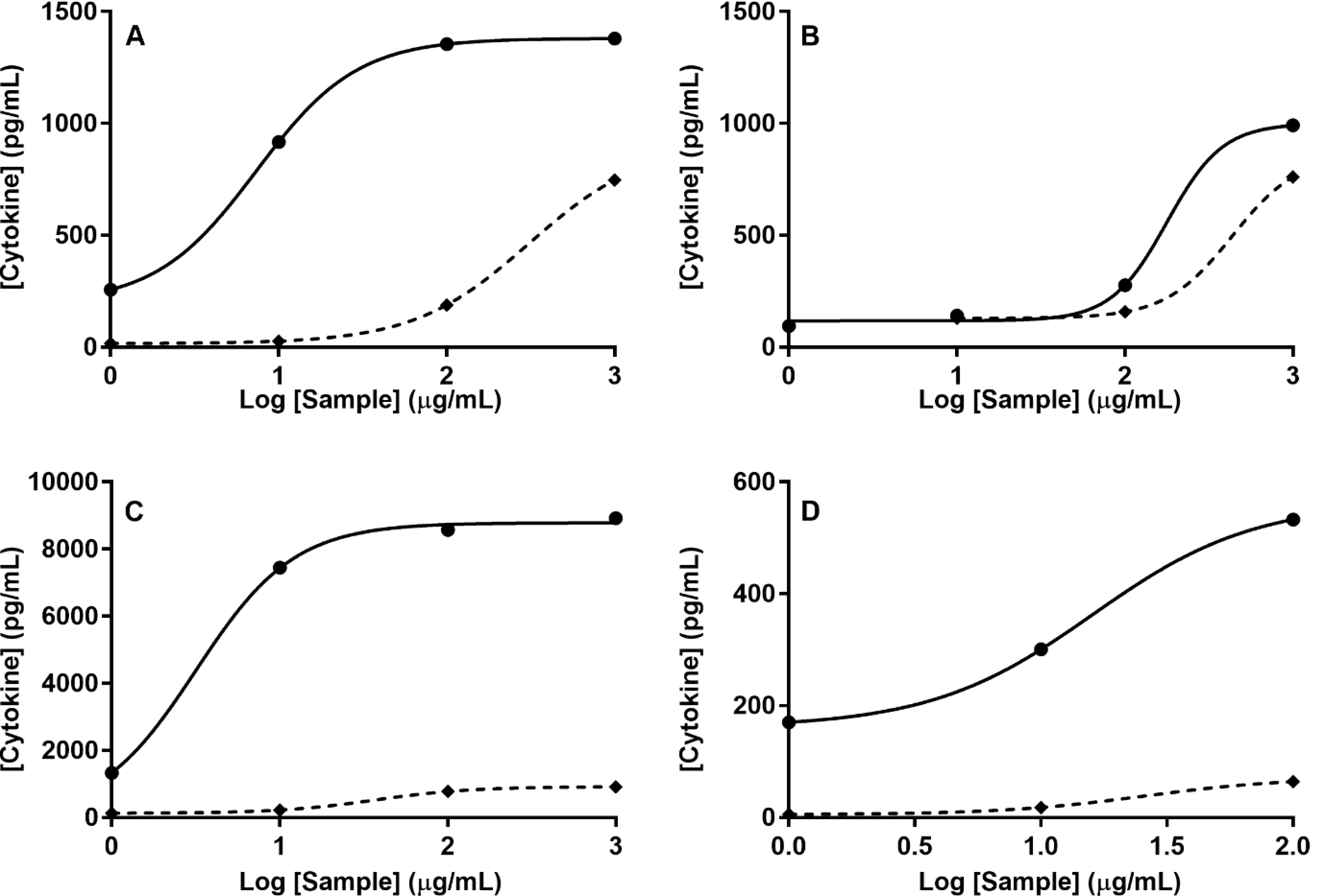

Supplement: S5 Fig — Concentrations are represented by log value and range from 1 to 1,000 μg/ml. Formula on LPS stimulated macrophages (black solid line), Formula on non-LPS stimulated macrophages (dashed line). (A) TNF-α, (B) IL-1α, (C) IL-6, (D) IL-10. (TIF) [file pone.0224740.s005.tif]

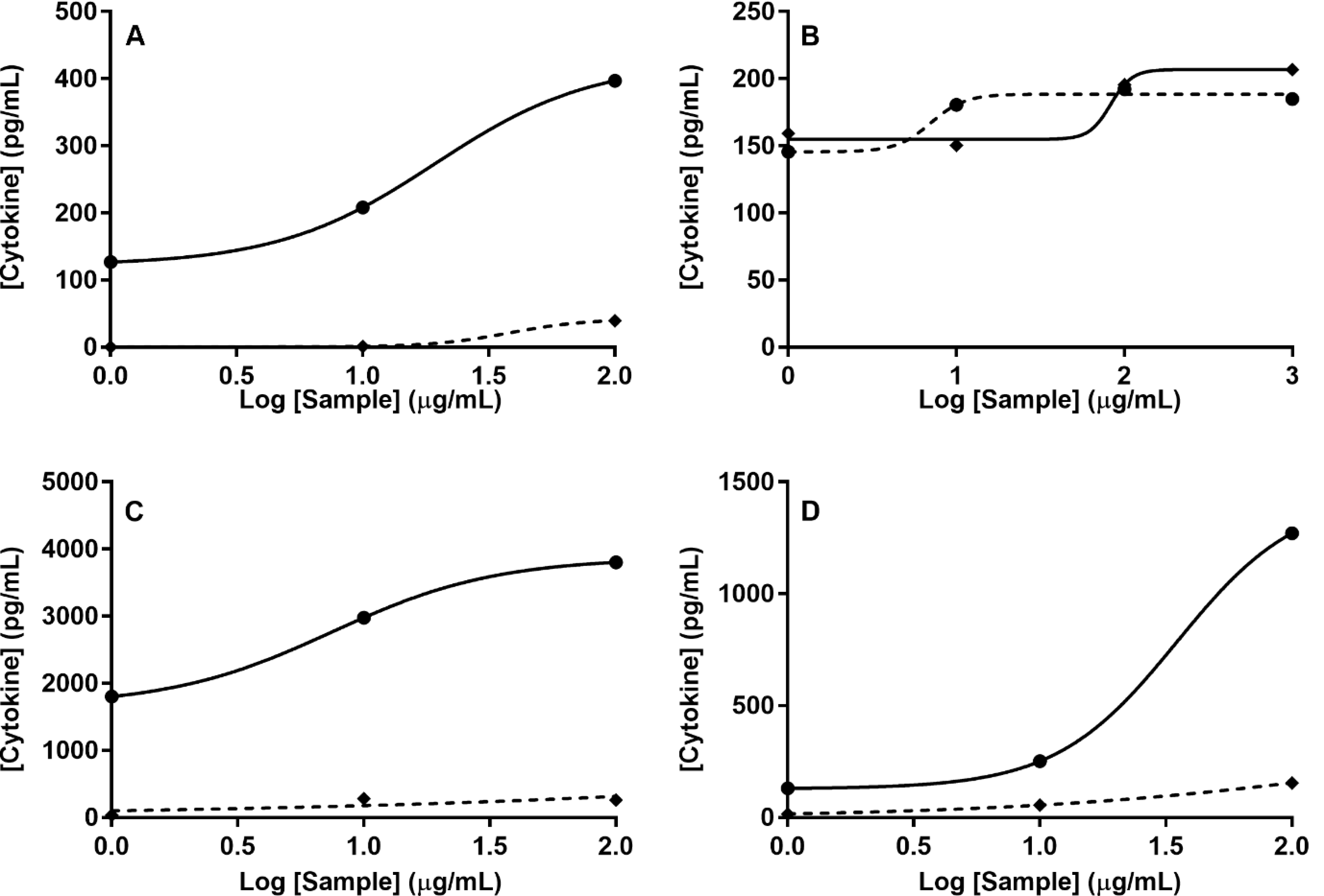

Supplement: S6 Fig — Concentrations are represented by log value and range from 1 to 1,000 μg/ml. Shiitake on LPS stimulated macrophages (black solid line), Shiitake on non-LPS stimulated macrophages (dashed line). (A) TNF-α, (B) IL-1α, (C) IL-6, (D) IL-10. (TIF) [file pone.0224740.s006.tif]

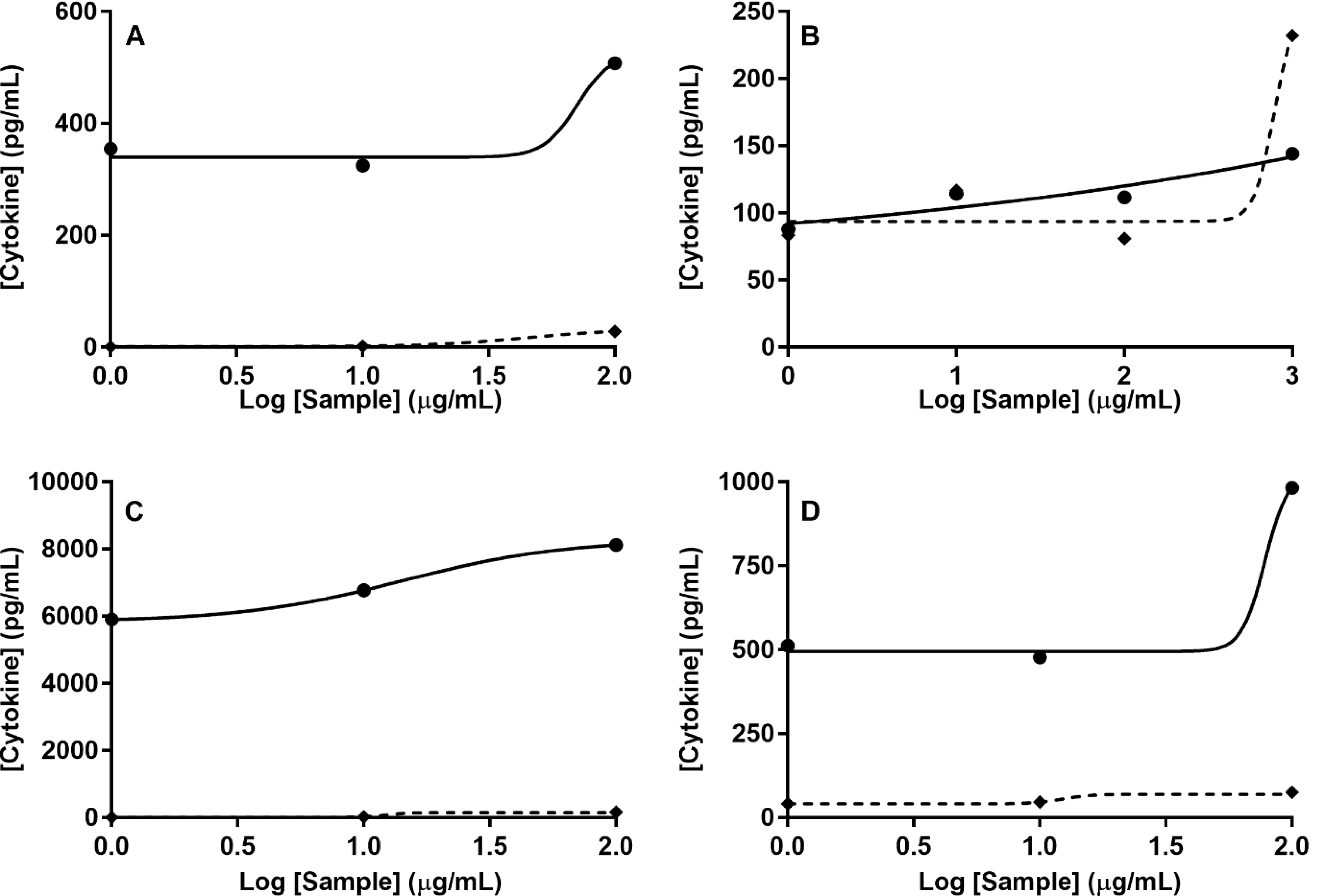

Supplement: S7 Fig — Concentrations are represented by log value and range from 1 to 1,000 μg/ml. Reishi on LPS stimulated macrophages (black solid line), Reishi on non-LPS stimulated macrophages (dashed line). (A) TNF-α, (B) IL-1α, (C) IL-6, (D) IL-10. (TIF) [file pone.0224740.s007.tif]

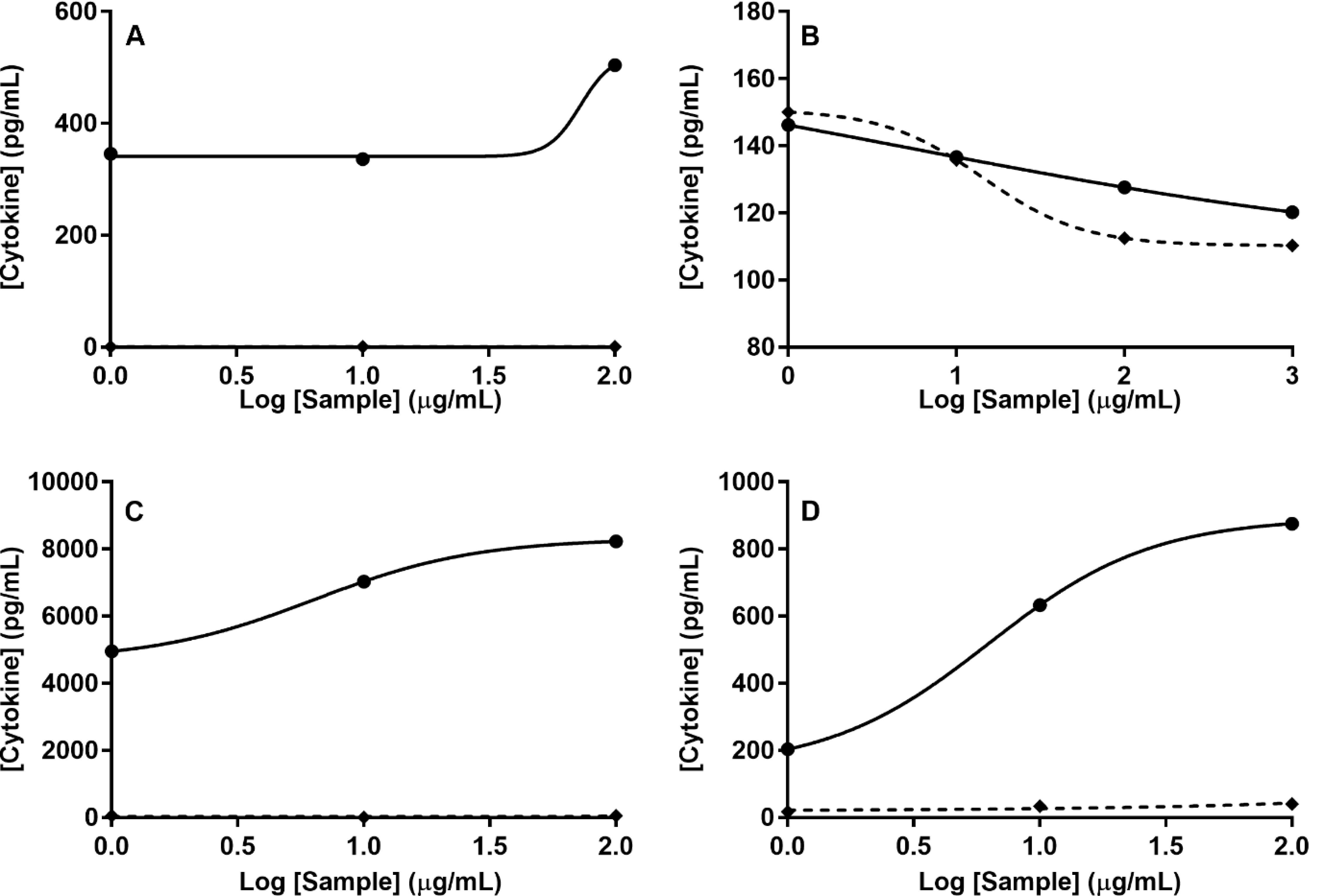

Supplement: S8 Fig — Concentrations are represented by log value and range from 1 to 1,000 μg/ml. Maitake on LPS stimulated macrophages (black solid line), Maitake on non-LPS stimulated macrophages (dashed line). (A) TNF-α, (B) IL-1α, (C) IL-6, (D) IL-10. (TIF) [file pone.0224740.s008.tif]

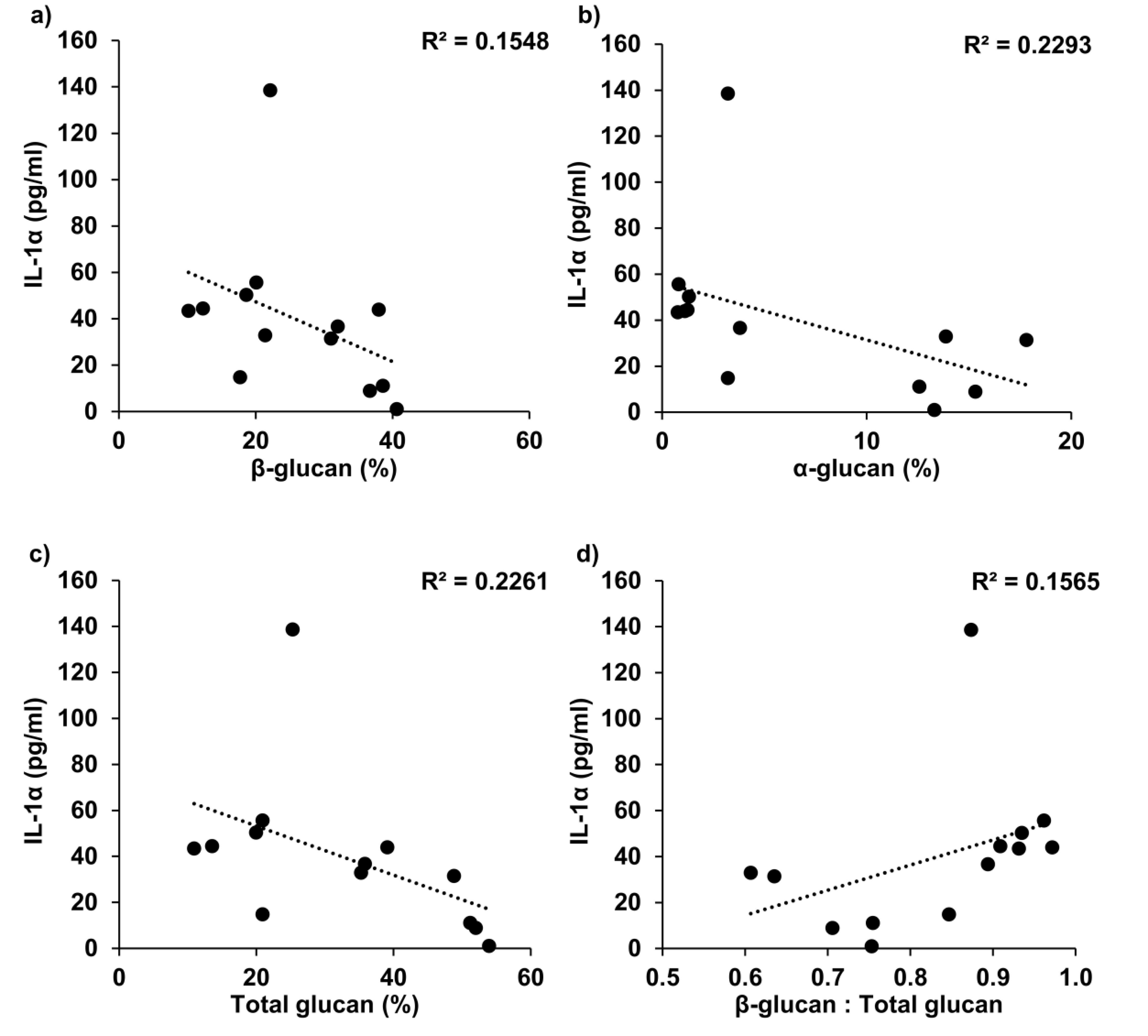

Supplement: S9 Fig — Expression levels versus percentage β-glucan (a), α-glucan (b), total glucan (c), β-glucan: total glucan ratio (d) on LPS non-stimulated macrophages following exposure to the mushroom extracts listed in Table 1. (TIF) [file pone.0224740.s009.tif]

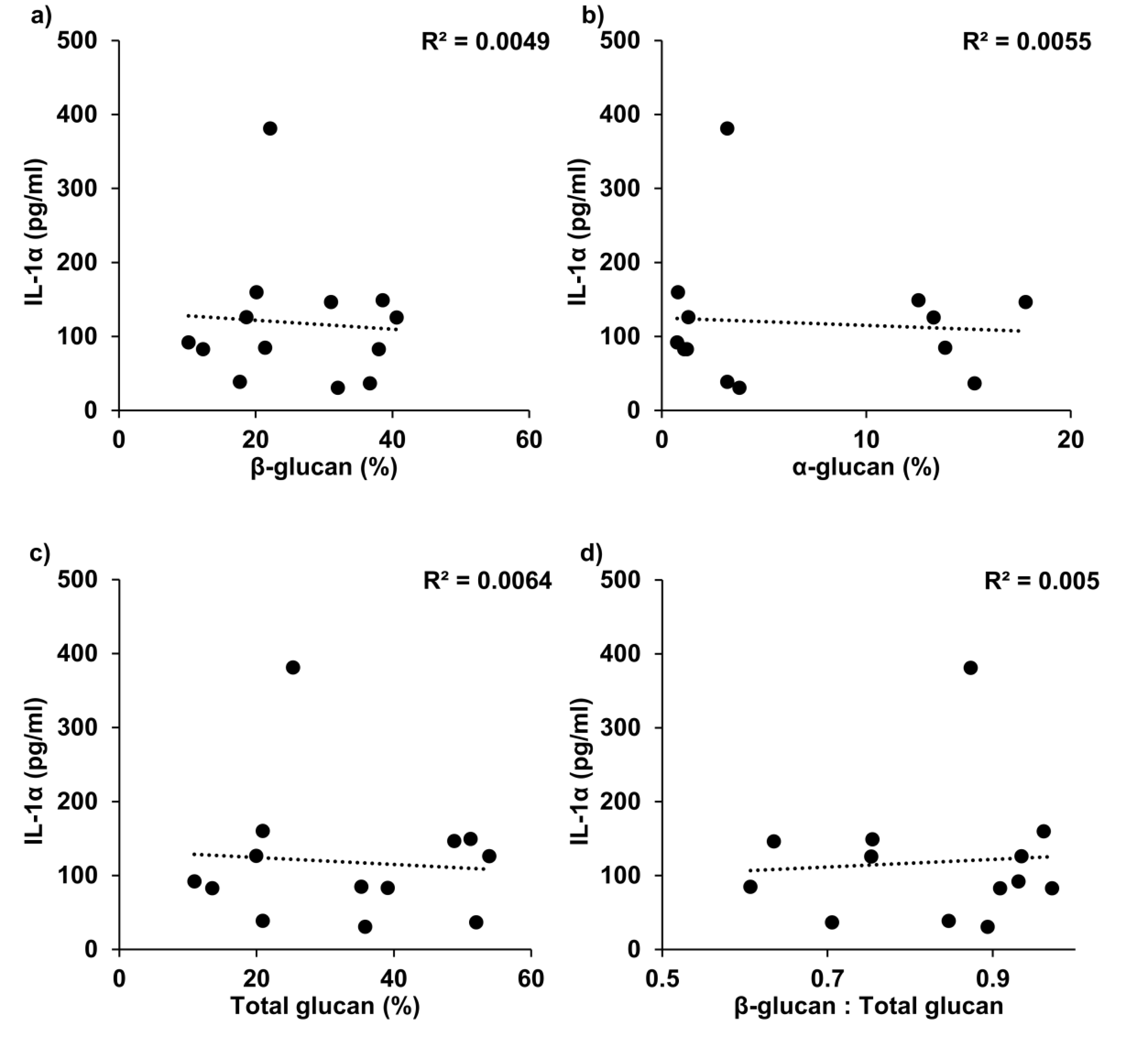

Supplement: S10 Fig — Expression levels versus percentage β-glucan (a), α-glucan (b), total glucan (c), β- glucan: total glucan ratio (d) on LPS stimulated macrophages following exposure to the mushroom extracts listed in Table 1. (TIF) [file pone.0224740.s010.tif]

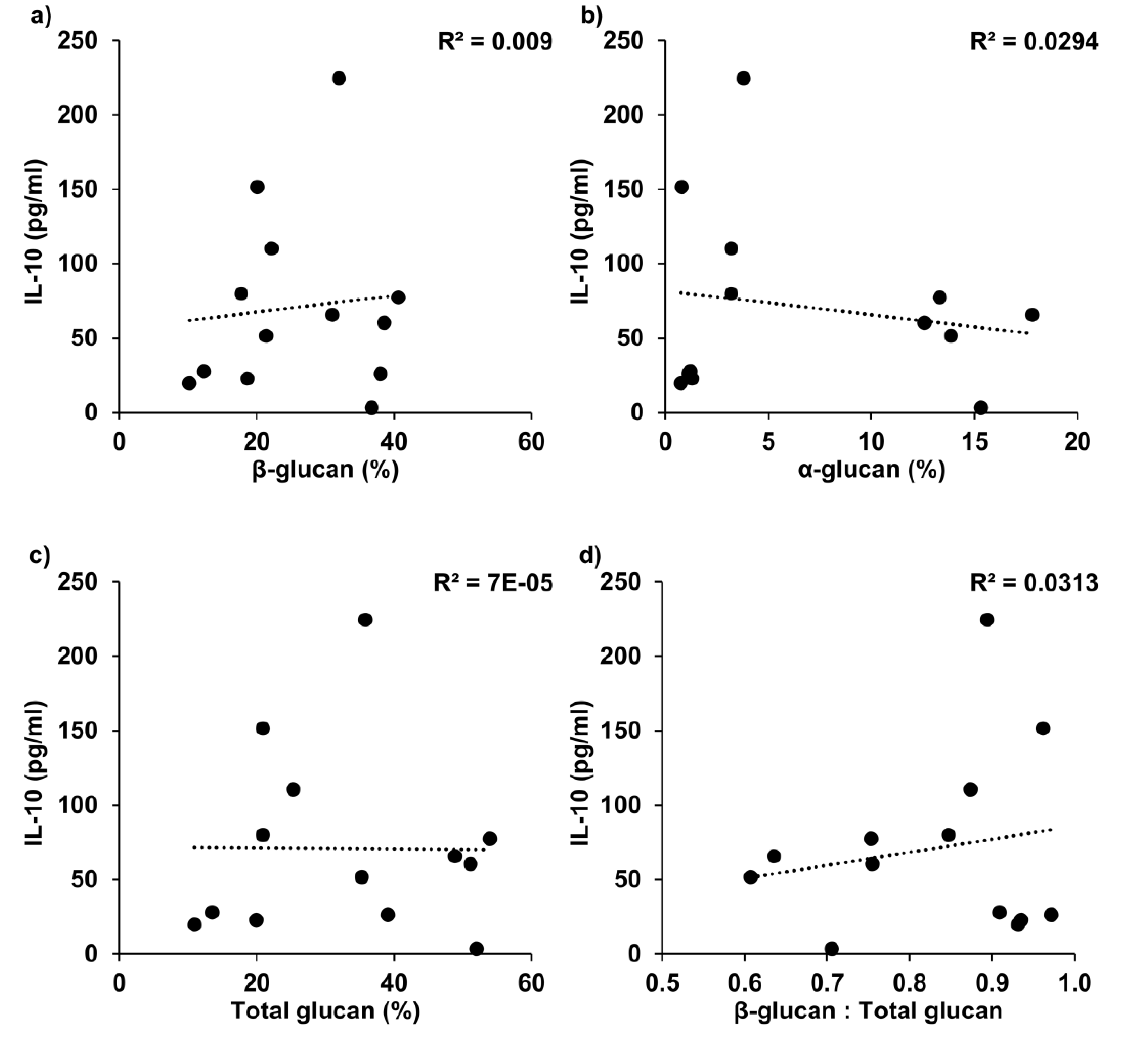

Supplement: S11 Fig — Expression levels versus percentage β-glucan (a), α-glucan (b), total glucan (c), β-glucan: total glucan ratio (d) on LPS non-stimulated macrophages following exposure to the mushroom extracts listed in Table 1. (TIF) [file pone.0224740.s011.tif]

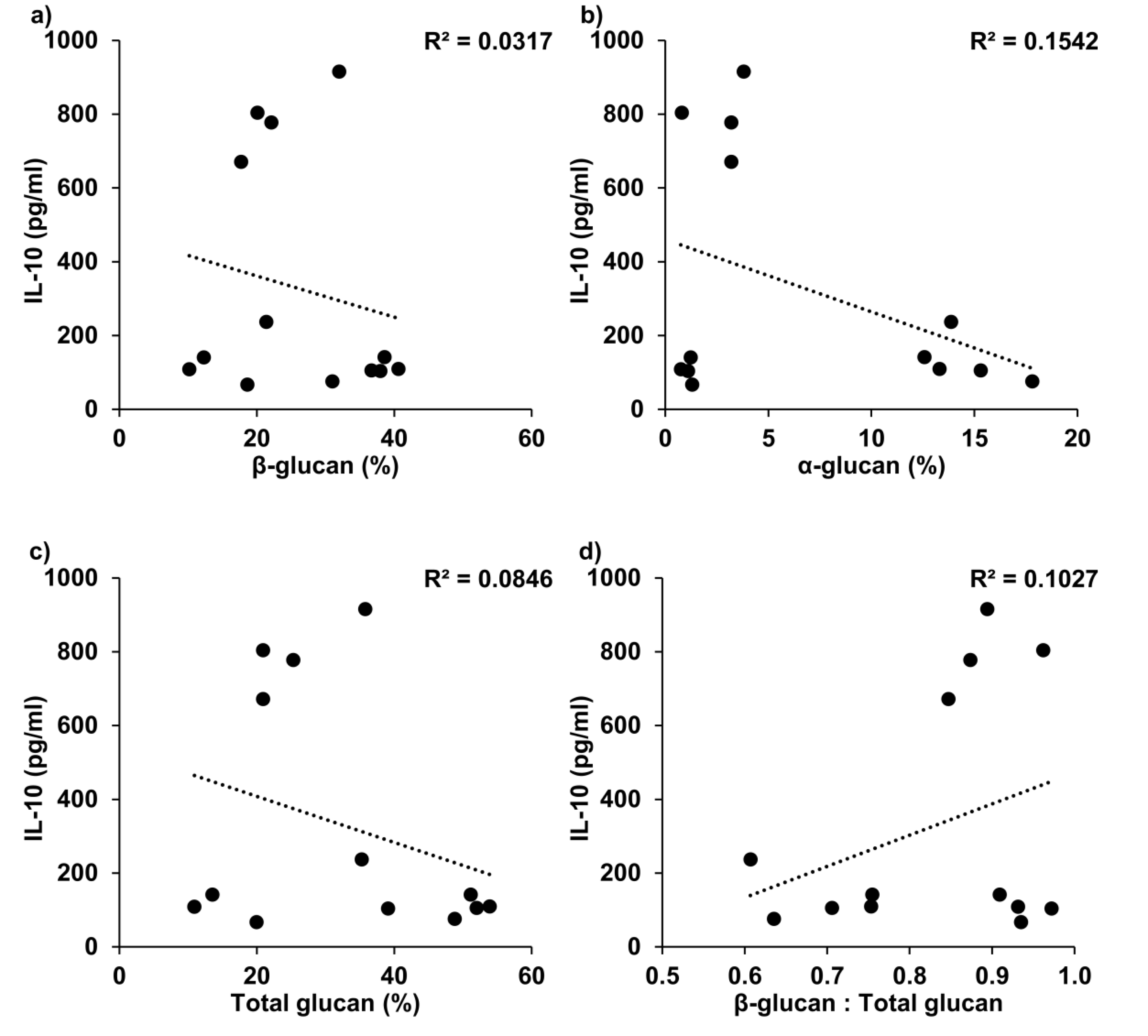

Supplement: S12 Fig — Expression levels versus percentage β-glucan (a), α-glucan (b), total glucan (c), β-glucan: total glucan ratio (d) on LPS stimulated macrophages following exposure to the mushroom extracts listed in Table 1. (TIF) [file pone.0224740.s012.tif]

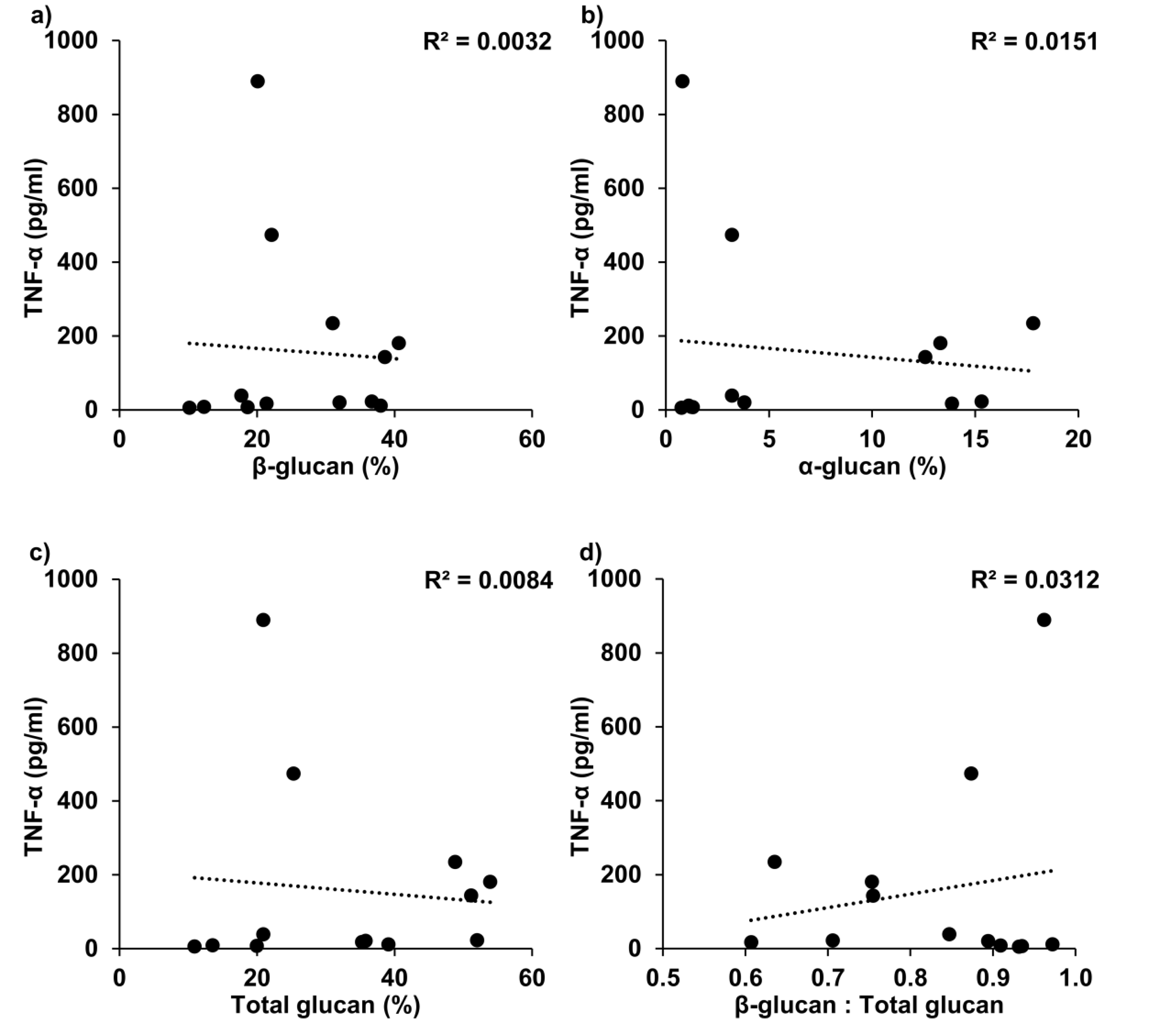

Supplement: S13 Fig — Expression levels versus percentage β-glucan (a), α-glucan (b), total glucan (c), β-glucan: total glucan ratio (d) on LPS non-stimulated macrophages following exposure to the mushroom extracts listed in Table 1. (TIF) [file pone.0224740.s013.tif]

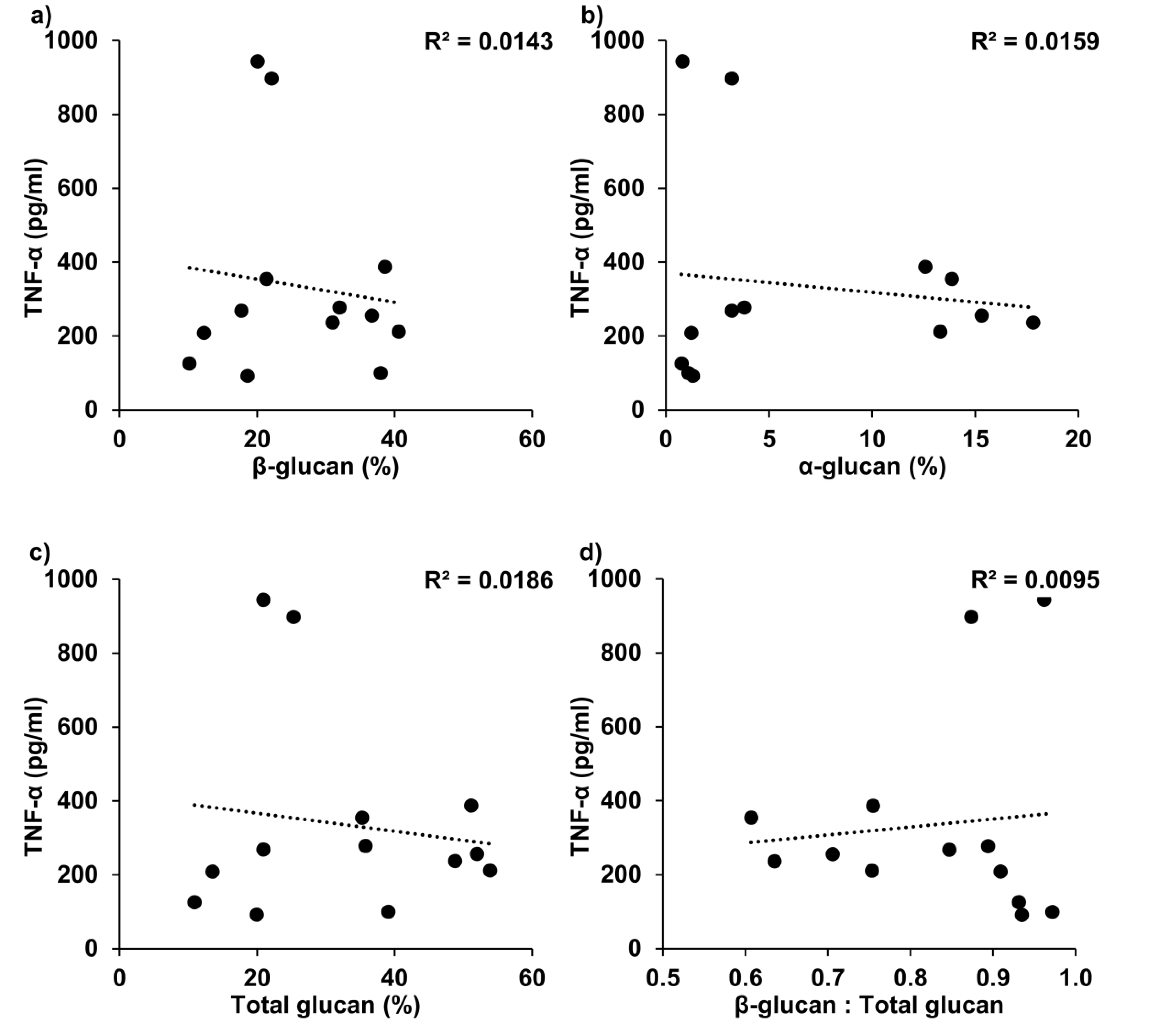

Supplement: S14 Fig — Expression levels versus percentage β-glucan (a), α-glucan (b), total glucan (c), β-glucan: total glucan ratio (d) on LPS stimulated macrophages following exposure to the mushroom extracts listed in Table 1. (TIF) [file pone.0224740.s014.tif]

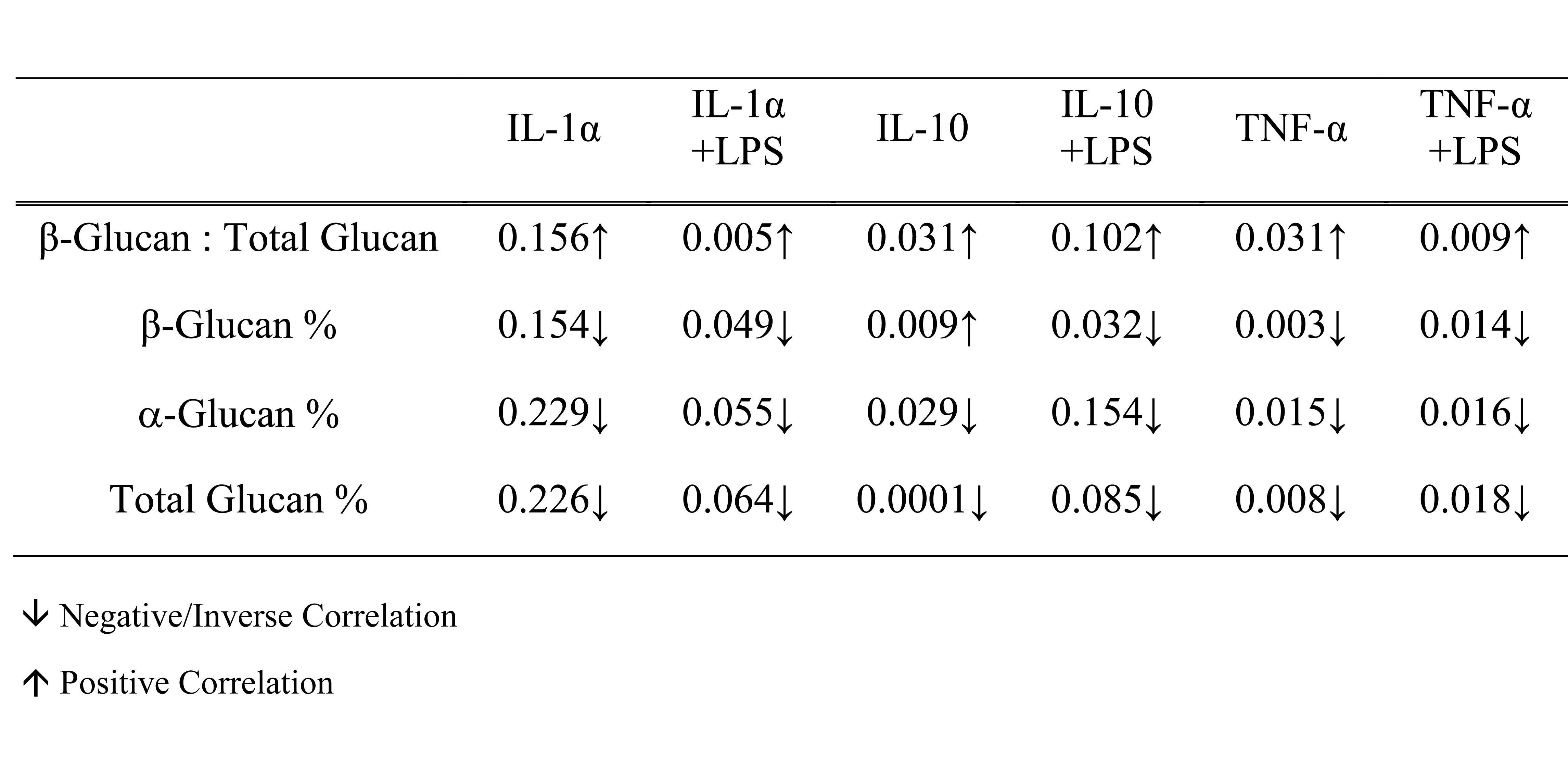

Supplement: S1 Table — R2 values for β-glucan, α-glucan, total glucan, and β-glucan: total glucan composition data between the mushroom preparations listed in Table 1 and cytokine expression in human macrophages ± LPS stimulation. (TIF) [file pone.0224740.s016.tif]
